# Supplementary material for: Dietary Intakes and Cardiovascular Health of Healthy Adults in Short-, Medium-, and Long-Term Whole-Food Plant-Based Lifestyle Program
Source: Nutrients. 2019 Dec 24;12(1):55. doi: 10.3390/nu12010055 (PMC7019440; doi:10.3390/nu12010055)
Supplement: Supplementary file 1 [file nutrients-12-00055-s001.pdf]

Table S1. Current demographic and other characteristics of study participants.

	Group 1 (n = 51)		Group 2 (n = 56)		Group 3 (n = 44)	
**Parameter**	**Female (n = 35)**	**Male (n = 16)**	**Female (n = 43)**	**Male (n = 13)**	**Female (n = 31)**	**Male (n = 13)**
**Partner status** (n (%))						
Married	14 (40)	4 (25.0)	23 (53.5)	2 (15.4)	13 (41.9)	3 (23.1)
Extramarital	10 (28.6)	6 (37.5)	23 (53.5)	9 (69.2)	12 (38.7)	7 (53.8)
Single	10 (28.6)	6 (37.5)	10 (23.3)	2 (15.4)	5 (16.1)	3 (23.1)
Widowed	1 (2.9)	0	0	0	1 (3.2)	0
**Living regions** (n (%))						
Capital city (Central)	17 (48.6)	9 (56.2)	9 (20.9)	4 (30.8)	11 (35.5)	7 (53.8)
Dolenjska (South East)	8 (22.9)	4 (25.0)	23 (53.5)	5 (38.5)	10 (32.3)	1 (7.7)
Gorenjska (North/North West)	3 (8.6)	1 (6.2)	4 (9.3)	2 (15.4)	3 (9.7)	3 (23.1)
Notranjska (Central/South West)	1 (2.9)	0	0	0	1 (3.2)	1 (7.7)
Prekmurje (North East)	1 (2.9)	0	0	0	0	0
Štajerska (East)	5 (14.3)	2 (12.5)	7 (16.3)	2 (15.4)	6 (19.4)	1 (7.7)
**Living environment** (n (%))						
City	7 (20)	7 (43.8)	10 (23.3)	3 (23.1)	8 (25.8)	6 (46.2)
Suburban	9 (25.7)	4 (25.0)	16 (37.2)	3 (23.1)	11 (35.5)	5 (38.5)
Rural	19 (54.3)	5 (31.2)	17 (39.5)	7 (53.8)	12 (38.7)	2 (15.4)
**Education** (n (%))						
Primary School/High School	14 (40.0)	11 (68.7)	13 (30.2)	4 (30.8)	10 (32.3)	5 (38.5)
College	4 (11.4)	0	5 (11.6)	4 (30.8)	2 (6.5)	1 (7.7)
Bachelor/University degree	15 (42.8)	5 (31.2)	23 (53.5)	5 (38.5)	18 (58.0)	6 (46.2)
Masters/PhD degree	1 (2.9)	0	2 (4.7)	0	1 (3.2)	1 (7.7)
N/A (did not disclose)	1 (2.9)	0	0	0	0	0
**Employment status** (n (%))						
Employed/help in family business	23 (65.7)	7 (43.8)	31 (74.4)	8 (61.5)	16 (51.6)	8 (61.5)
Self-employed/contract	3 (8.6)	2 (12.5)	5 (11.6)	4 (30.8)	9 (29.0)	4 (30.8)
Retired	1 (2.9)	1 (6.2)	2 (4.6)	1 (7.7)	2 (6.5)	0
High-school/student	7 (20.0)	5 (31.2)	1 (2.3)	0	2 (6.5)	1 (7.7)
Unemployed	1 (2.9)	1 (6.2)	3 (7.0)	0	2 (6.5)	0
**Economic status** (n (%))						
Low income/financially challenged	3 (8.6)	1 (6.2)	2 (4.7)	1 (7.7)	0	1 (7.7)
Average/mildly above average	31 (88.5)	14 (87.5)	40 (93.0)	10 (76.9)	30 (96.8)	12 (92.3)

	Group 1 (n = 51)		Group 2 (n = 56)		Group 3 (n = 44)	
**Parameter**	**Female (n = 35)**	**Male (n = 16)**	**Female (n = 43)**	**Male (n = 13)**	**Female (n = 31)**	**Male (n = 13)**
Above average	1 (2.9)	1 (6.2)	1 (2.3)	2 (15.4)	1 (3.2)	0
**Income** (€ (%))						
Up to 700	6 (17.1)	1 (6.7)	2 (4.6)	0	0	0
701–1100	4 (11.5)	3 (20.0)	3 (7.0)	0	2 (6.4)	1 (7.7)
1101–1500	5 (14.3)	1 (6.7)	7 (16.3)	1 (7.7)	3 (9.7)	2 (15.4)
1501–1900	11 (31.4)	2 (13.3)	8 (18.6)	4 (30.8)	2 (6.4)	4 (30.8)
From 1901	9 (25.7)	8 (53.3)	23 (53.5)	8 (61.5)	23 (74.2)	6 (46.1)
N/A (did not disclose)	0	0	0	0	1 (3.2)	0
**Smoking** (n (%))						
Current	4 (11.4)	3 (18.8)	7 (16.7)	2 (15.4)	8 (25.8)	2 (15.4)
Former	3 (8.6)	1 (6.2)	2 (4.8)	0	1 (3.2)	0
Never	28 (80.0)	12 (75.0)	33 (78.6)	11 (84.6)	22 (71.0)	11 (84.6)
**Menstrual status** (n (%))						
Menstruation	29 (82.9)	-	32 (74.4)	-	23 (74.2)	-
Perimenopause	2 (5.7)	-	0	-	2 (6.5)	-
Menopause	4 (11.4)	-	11 (25.6)	-	6 (19.4)	-
**Medications** (n)						
Thyroid medication	1	0	1	0	0	0
Birth-control pills	1	0	1	0	0	0
Vitamin D_3 prescribed by doctor‡	2	1	1	0	1	1
Nausea	0	0	1	0	0	0
Others	1	0	1	0	0	0

Data are means (standard deviation). ‡Vitamin D_3 here represents only medically prescribed vitamin D.

Table S2. Dietary intake of energy and macronutrients of all participants in WFPB lifestyle program (0.5-10 years) according to genders comparison.

	Whole sample (n = 151)		
	Female (n = 109)	Male (n = 42)	*p*-Values
**Macronutrients (per day)**			F vs. M
Energy intake (kcal)	1841 (539)	2618 (726)	<0.001
Carbohydrates (g)	266 (87)	370 (98)	<0.001
(% E)	58 (5)	57 (6)	0.354
Total sugars (g)	80 (35)	101 (37)	<0.001
(% E)	17 (4)	16 (4)	0.028
Free sugars (g)	22 (18)	24 (11)	0.007
(% E)	5 (3)	4 (2)	0.177
Starches (g)	82 (45)	128 (52)	<0.001
(% E)	18 (7)	19 (6)	0.110
Dietary fibre (g)	64 (16)	85 (23)	<0.001
(% E)	7.1 (1)	6.6 (1)	0.015
Fat (g)	40 (16)	63 (31)	<0.001
(% E)	20 (5)	21 (6)	0.252
SFA (g)	6 (3)	10 (4)	<0.001
(% E)	3 (1)	3 (2)	0.051
MUFA (g)	11 (5)	18 (10)	<0.001
(% E)	5 (2)	6 (2)	0.125
PUFA (g)	18 (7)	28 (15)	<0.001
(% E)	9 (3)	9 (3)	0.337
Linoleic acid (g)	11 (6)	19 (11)	<0.001
Alpha-linolenic acid (g)	5 (3)	7 (4)	0.005
EPA (mg)	332 (233)	338 (183)	0.630
DHA (mg)	226 (163)	245 (156)	0.582
ARA (mg)	2 (7)	3 (9)	0.819
Cholesterol (mg)	6 (18)	10 (18)	0.459
Protein (g)	70 (19)	100 (26)	<0.001
(% E)	15 (2)	15 (3)	0.891
(g/kg body mass)	1.1 (0.4)	1.3 (0.4)	0.063
Water [w] (L)	3.9 (1.2)	4.2 (1.2)	0.063

Data are means (standard deviation). [w] Water from foods and beverages. A T-test was used to compare differences between genders.

Table S3. Dietary intake of selected vitamins, minerals, and trace minerals of all participants in WFPB lifestyle program (0.5-10 years) according to genders comparison.

	Whole sample (n = 151)		
**Micronutrients (per day)**	Female (n = 109)	Male (n = 42)	*p*-Values
**Vitamins**			F vs. M
Thiamine (mg)	2.9 (1.4)	3.4 (1.0)	<0.001
Riboflavin (mg)	3.0 (1.8)	2.9 (1.6)	0.797
Niacin (mg)	30 (3)	34 (10)	0.003
Pantothenic acid (mg)	11 (3)	12 (3)	0.043
Vitamin B_6 (pyridoxine) (mg)	3.9 (1.3)	4.5 (1.1)	0.002
Biotin (μg)	100 (40)	110 (43)	0.138
Folate (μg folate equivalent)	763 (211)	929 (285)	<0.001
Vitamin B_{12} (μg)	247 (320)	337 (379)	0.056
Vitamin C (mg)	334 (164)	344 (146)	0.715
Retinol equivalents[re] (mg)	3.6 (2.0)	4.6 (3.0)	0.012
Vitamin D (μg)	11 (11)	9 (7)	0.157
Vitamin E (mg)	28 (12)	31 (25)	0.881
**Minerals**			
Calcium (mg)	1016 (292)	1251 (362)	<0.001
Magnesium (mg)	839 (293)	1042 (328)	<0.001
Phosphorus (mg)	1663 (411)	2233 (584)	<0.001
Potassium (mg)	4554 (1278)	5915 (1577)	<0.001
Sodium (mg)	1955 (933)	2270 (1052)	0.073
Chloride (mg)	3160 (1453)	3684 (1616)	0.037
**Trace elements**			
Iron (mg)	35 (19)	40 (10)	<0.001
Copper (mg)	3.8 (1.1)	4.9 (1.6)	<0.001
Iodine (μg)	230 (75)	247 (71)	0.139
Zinc (mg)	19 (5)	23 (6)	<0.001
Chrome (μg)	80 (31)	77 (34)	0.520
Molybdenum (μg)	94 (63)	85 (59)	0.282
Selenium (μg)	112 (44)	128 (46)	0.028

Data are means (standard deviation). [re]Retinol equivalents = vitamin A + α-carotene (1 mg retinol equivalent = 12 mg α-carotene) + β-carotene (1 mg retinol equivalent = 6 mg β-carotene) + γ-carotene (1 mg retinol equivalent = 12 mg γ-carotene*).* A T-test was used to compare differences between genders.

Table S4. Cardiovascular health (lipids and BP) and safety marker status (serum UA and haemoglobin concentration) for all participants in WFPB lifestyle program (0.5-10 years) according to gender comparison.

**Parameter**	**Whole sample**		**_p_-Value**
**Gender (F/M)**	Female (n = 109)	Male (n = 42)	F vs. M
**Laboratory variables**			
S-cholesterol (mmol/L)	3.7 (0.8)		
	3.8 (0.7)	3.4 (0.9)	0.002
LDL-cholesterol (mmol/L)	2.0 (0.7)		
	2.0 (0.6)	2.0 (0.7)	0.451
HDL-cholesterol (mmol/L)	1.4 (0.4)		
	1.5 (0.3)	1.1 (0.2)	<0.001
Triglycerides (mmol/L)	0.9 (0.4)		
	0.8 (0.3)	1.0 (0.4)	0.037
S-Uric Acid (μmol/L)	273 (68)		
	245 (50)	346 (52)	<0.001

Haemoglobin (g/L)	141 (12)			
		137 (10)	153 (9)	<0.001
**Blood pressure (mmHg)**				
Systolic	115 (11)			
		113 (11)	120 (10)	<0.001
Diastolic	71 (9)			
		71 (9)	72 (8)	0.143

Data are means (standard deviation). A T-test was used to compare differences between genders.
